# Investigating the Relationship Between Body Constitution as Defined Under Traditional Chinese Medicine and Vertigo in Women in Taiwan

**DOI:** 10.1097/jnr.0000000000000718

**Published:** 2026-01-09

**Authors:** Pei-Ling TANG, Hsueh-Chih CHOU, Miao-Ling LIN, Te-Fang WU, Tzu-Cheng PAN

**Affiliations:** 1 Department of Nursing, School of Nursing, Fooyin University, Kaohsiung City, Taiwan, ROC; 2 Department of Nursing, Kaohsiung Veterans General Hospital, Kaohsiung City, Taiwan, ROC; 3 Department of Nursing, Shu-Zen Junior College of Medicine and Management, Kaohsiung City, Taiwan, ROC; 4 Department of Business Management, National Sun Yat-Sen University, Kaohsiung City, Taiwan, ROC; 5 Department of Otorhinolaryngology, Kaohsiung Veterans General Hospital, Kaohsiung City, Taiwan, ROC; 6 Department of Orthopedics, Kaohsiung Veterans General Hospital, Kaohsiung City, Taiwan, ROC

**Keywords:** body constitution, female, Traditional Chinese Medicine, vertigo, women’s health

## Abstract

**Background:**

Despite its status as a prevalent clinical concern, vertigo lacks an effective treatment, especially one that addresses the factors commonly associated with women affected by this condition.

**Purpose:**

This study was designed to clarify the relationship between Traditional Chinese Medicine Body Constitution (Yang-Xu, Yin-Xu, and Phlegm stasis) and vertigo in adult women.

**Methods:**

A cross-sectional study was conducted on 1,423 women enrolled in the Taiwan Biobank between 2012 and 2017. The participants completed the 44-question Body Constitution Questionnaire (BCQ), developed in Taiwan, to assess their body constitution. To investigate the relationship between body constitution and vertigo prevalence, a stepwise-forward multiple-regression analysis was conducted to investigate the influence of three specific body constitutions (Yang-Xu, Yin-Xu, and Phlegm stasis) on the risk of contracting vertigo.

**Results:**

Nearly one out of 10 (9.0%) participants had experienced vertigo, with an average onset age of 56.23 years, with vertigo sufferers showing a significantly higher mean BCQ score compared to those who had not experienced vertigo (Yang deficiency: adjusted Odds Ratio [a*OR*]=1.033, Yin deficiency: a*OR*=1.049, and Phlegm stasis: a*OR*=1.041). Age, having migraines, and having endometrial polyps were identified as factors influencing the risk of experiencing vertigo.

**Conclusions/Implications for Practice:**

Unbalanced constitution types were independently associated with a higher risk of vertigo in women, alongside age, migraine, and endometrial polyps. These findings suggest that the constitution serves as an important underlying susceptibility factor. Incorporating constitution assessment into routine health evaluation may facilitate earlier identification of at-risk women and provide a foundation for preventive or constitution-balancing strategies in clinical care.

## Introduction

Vertigo is a common clinical symptom associated with a positioning disorder in the human body that leads to sensations of spinning, bumpy walking, and an inability to balance and is often accompanied by symptoms such as nausea and vomiting (Dy & Freeman, 2025). Some 1.8% of the general population has sought medical attention for sudden or recurrent vertigo, and 15%–20% suffer from vertigo symptoms, with this condition more common in women than men (Neuhauser, 2016). A study by Kim et al. (2020) on 21,166 patients with vertigo reported a difference in the incidence of vertigo between men and women. In addition to differences in physiological structure (e.g., presence of the uterus and ovaries) and functions, women experience endocrine fluctuations associated with menstruation, pregnancy, and menopause (P.-H. Chen et al., 2020; S.-L. Chen et al., 2021).

Although vertigo does not increase risk of death, it is difficult to treat and causes physical and psychological distress that reduces quality of life (Agrawal et al., 2018; Neuhauser, 2016; J. Wang et al., 2021). Moreover, frequent and severe recurrent or chronic vertigo greatly interferes with the ability to work. An earlier study showed that 27% of vertigo sufferers changed jobs, 21% gave up work, and 50% experienced a reduction in their work efficiency (Bronstein et al., 2010). In addition, vertigo can cause or exacerbate mental problems, and anxiety over vertigo has been shown to impair social life (Kovacs et al., 2019). Vertigo patients often receive multiple treatments to improve their condition. Western medical treatment relies mainly on medications, including antivertigo and antiemetic drugs. Also, 41.3% of these patients receive physical therapy (Grill et al., 2014), although differences in diagnostic measures, treatment methods, and medications reflect a lack of standardization in treatment and prevention methods (Tsai et al., 2016). Concurrently, interest has been growing in the use of Traditional Chinese Medicine (TCM) as a complementary approach for managing vertigo, given its potential to relieve symptoms and support recovery (Tsai et al., 2016).

A fundamental tenet of TCM is that health is achieved and maintained through the harmonious balance of Yin and Yang and that any imbalance between the two may result in ill health and/or disease onset. TCM is widely used in Asia, and treatment methodologies such as Taiwan’s TCM constitution (L.-L. Chen et al., 2009; J.-D. Lin, Lin, et al., 2012; J.-S. Lin et al., 2012), Sasang constitutional medicine from Korea (Son et al., 2015), Traditional Japanese Kampo Medicine (Watanabe et al., 2011), and Vietnamese Traditional Medicine (Thompson, 2015) are all noteworthy. TCM emphasizes the importance of personalized medicine based on the body constitution of each individual (Li et al., 2019) as determined by the yin-yang state of the body and influenced by factors such as environment, age, lifestyle, and diet (Li et al., 2019). Yin reflects physiological function (including blood, body fluids, and jin ye), while yang reflects the energies that maintain physiological function (including digestion, heart contraction, and respiration). Phlegm stasis is induced when the transport of energy substances such as blood sugar and glycosylated hemoglobin is obstructed by external or environmental stimuli (L.-L. Chen et al., 2009; J.-D. Lin, Lin, et al., 2012; J.-S. Lin et al., 2012). The body constitution may be balanced, which is considered a healthy physical state, or unbalanced, which may reflect either Yang-Xu, Yin-Xu, or Phlegm stasis and makes individuals more susceptible to certain diseases (Z. Guo et al., 2017; Ma et al., 2021). The acupuncture points used to treat vertigo are located in the upper orifices, and vertigo is caused by internal issues such as an increase in liver yang, deficiencies of qi and blood, and internal dampness and turbidity (Z. Guo et al., 2017). To treat vertigo, the flow of qi and blood must be promoted in the three viscera (liver, kidney, and spleen; Z. Guo et al., 2017; Ma et al., 2021).

In light of the influence of the constitution on treatment modality, accurately classifying body constitution in each patient is crucial. Moreover, clarifying the association between the female body constitution and vertigo is necessary to optimize treatment effectiveness and safety (P.-H. Chen et al., 2022). Based on the above, the aim of this study was to investigate the connection between unbalanced constitution types (Yang-Xu, Yin-Xu, and Phlegm stasis) and vertigo in adult women in Taiwan, with the findings expected to provide valuable insights for personalized preventive medicine.

## Methods

### Data Source

The Taiwan Biobank (TWB) is a database of longitudinal health status information on individuals living in Taiwan that includes questionnaire, physical examination result, specimen value, lifestyle pattern, dietary habit, and environmental factor data (Taiwan Biobank, 2020). The TWB has been widely used in studies involving body constitution, women’s issues, and chronic diseases (P.-H. Chen et al., 2020; S.-L. Chen et al., 2021). TWB is Taiwan’s first government-commissioned population database that provides controlled data access for academic research (Taiwan Biobank, 2020).

### Study Design and Participants

**Figure 1:**
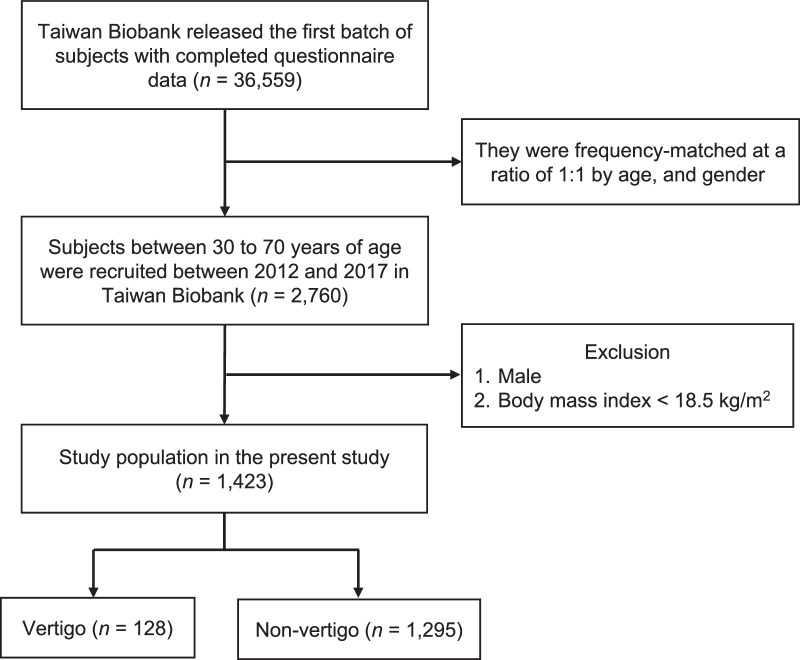
The Flowchart Diagram of Subjects’ Selections

In this cross-sectional study, data from 1,423 women aged between 30 and 70 years extracted from the 36,559 participants in the first TWB batch between 2012 and 2017 were used to investigate the association between body constitution (Yang-Xu, Yin-Xu, and Phlegm stasis) and vertigo in women. The sample was obtained after excluding all men in the database and all women with a body mass index (BMI)<18.5 kg/m^2^. Health-related characteristics and body constitution questionnaire data were fully included (Figure 1). Vertigo status was based on a self-reported questionnaire that included questions related to vertigo, such as “Were you ever diagnosed with vertigo?”

### Measurements

The questionnaire data used in this study included:

1. Sociodemographic characteristics: age, BMI, marital status, living status, educational level, work status, metabolic syndrome (hypertension, hyperlipidemia, and diabetes), and pain (lower back pain and migraines).
2. Health-related characteristics: regularity of menstrual cycles, menstruation status, period pain, uterine myomas, ovarian cysts, endometriosis, endometrial polyps, previous use of hormone medications for ≥6 months, previous use of women’s health-conditioning TCM products for ≥3 years, and previous use of women’s health supplements for ≥3 months.
3. Body Constitution Questionnaire (BCQ): The BCQ was developed by a research team in Taiwan to assess body constitution type and syndromes (L.-L. Chen et al., 2009; J.-D. Lin, Lin, et al., 2012; J.-S. Lin et al., 2012). In 2007, the BCQ was recognized as a national standard measure that could be applied in clinical trials using TCM interventions. The 44 items of the BCQ are scored on a 5-point Likert scale rating either intensity or frequency. Some items appear in more than one constitution category because certain symptoms may correspond to multiple constitutional imbalances in TCM theory. For each constitution type (Yang-Xu, Yin-Xu, and Phlegm stasis), scores are summed separately, and the participant’s predominant constitution is determined according to the established cutoff thresholds for each category.

The criteria for each of the three body constitution types are Yang-Xu—total score of 19 Yang-Xu items ≥31; Yin-Xu—total score of 19 Yin-Xu items ≥30; and Phlegm stasis—total score of 16 Phlegm stasis items ≥27. Higher scores indicate higher deviation from the body constitution standard. Respondents who meet the criteria for two or more body constitutions are classified as having a “mixed” constitution. Respondents who meet the criteria for none of the three are classified as “balanced.” This scale distinguishes between mixed and balanced types and cannot assess the degree/severity. The content validity of all BCQ items was ≥.7, as evaluated using a Delphi process (L.-L. Chen et al., 2009; J.-D. Lin, Lin et al., 2012; J.-S. Lin et al., 2012). Cronbach’s α coefficients and intraclass correlation coefficients for the three body constitution types were Yang-Xu: .88 and .91 (L.-L. Chen et al., 2009; Su et al., 2008), Yin-Xu: .85 and .91 (J.-D. Lin, Chen, et al., 2012; J.-S. Lin et al., 2012), and phlegm stasis: .88 and .91 (J.-D. Lin, Lin, et al., 2012), respectively.

### Statistical Analysis

All of the statistical analyses were performed using SPSS Statistics Version 22.0 (IBM Corp., Armonk, NY, USA). Categorical variables were compared using the Pearson χ^2^ and expressed as numbers and percentages, while continuous variables were compared using an independent *t* test and expressed as mean and *SD*. To explore the influence of the three BCQ scores on women with vertigo, the stepwise-forward method was used in the multiple logistic regression analysis, and the results of the analysis were used to identify the most predictive factors. The model was adjusted for three BCQ constitution score categories, sociodemographic variables, and women’s health-related characteristics, with a *p* value of .05 considered statistically significant.

### Ethical Approval

The TWB recruitment process complied with regulations and ethics, guaranteed the management of personal privacy information in the database, and included an informed consent procedure that clearly explained participants’ rights, including voluntary participation and the right to withdraw their data at any stage. This study was approved by the institutional review board (VGHKS18-CT6-03), and all methods were performed in accordance with relevant guidelines and regulations (Declaration of Helsinki).

## Results

Of the 1,423 women included in the study sample, 128 (9.0%) had vertigo. The mean age of the sample was 52.81 years (*SD*=9.02 years) and the mean BMI was 24.09 kg/m^2^ (*SD*=3.01 kg/m^2^). The correlations between vertigo and sociodemographic characteristics in the sample are shown in Table 1. Compared to those without vertigo, those with vertigo tended to be older (mean ± *SD*: 52.48±8.98 years vs. 56.23 years, *p*<.001), live alone (7.4% vs. 12.5%, *p*=.041), not work (39.5% vs. 49.2%, *p*=.033), have hyperlipidemia (9.3% vs. 16.4%, *p*=.010), and suffer from lower back pain (33.1% vs. 43.0%, *p*=.024) and migraines (25.5% vs. 35.2%, *p*=.018).

**Table 1:** Comparison of Baseline Sociodemographic Characteristics in Patients With and Without Vertigo

Characteristic	Total (*n*=1,423)	Vertigo (*n*=128, 9%)	Non-vertigo (*n*=1,295, 91%)	*p*
	*n* (%)	*n* (%)	*n* (%)	
Age (years; *M* and *SD*)	52.81±9.02	56.23±8.82	52.48±8.98	<.001 ^a^
BMI (kg/m^2^; *M* and *SD*)	24.09±3.01	24.19±2.75	24.08±3.03	.669 ^a^
Marital status				.518
Unmarried	334 (23.5)	33 (25.8)	301 (23.2)	
Married	1,089 (76.6)	95 (74.2)	994 (76.8)	
Living status				.041
Not alone	1,311 (92.1)	112 (87.5)	1,199 (92.6)	
Alone	112 (7.9)	16 (12.5)	96 (7.4)	
Educational level				.218
Primary or middle school	524 (36.8)	56 (43.8)	468 (36.1)	
High school (vocational)	357 (25.1)	27 (21.1)	330 (25.5)	
College or above ^b^	542 (38.1)	45 (35.2)	497 (38.4)	
Employed				.033
Yes	848 (59.6)	65 (50.8)	783 (60.5)	
No	575 (40.4)	63 (49.2)	512 (39.5)	
Metabolic syndrome				
Hyperlipidemia, yes	141 (9.9)	21 (16.4)	120 (9.3)	.010
Hypertension, yes	168 (11.8)	19 (14.8)	149 (11.5)	.264
Diabetes, yes	66 (4.6)	12 (9.4)	54 (4.2)	.008
Low back pain				.024
No	940 (66.1)	73 (57.0)	867 (66.9)	
Yes	483 (33.9)	55 (43.0)	428 (33.1)	
Migraine				.018
No	1,048 (73.6)	83 (64.8)	965 (74.5)	
Yes	375 (26.4)	45 (35.2)	330 (25.5)	

*Note.* BMI=body mass index.

^a^ Independent *t* test.

^b^ College or post-graduate education.

More than 80% of the participants had irregular menstrual cycles, less than half were still menstruating (40.8%), a few experienced menstrual pain (5.0%), 4%–27% had one or more gynecological conditions, including uterine fibroids, ovarian cysts, endometriosis, and endometrial polyps, and 8%–15% used women-related supplements (hormone medications, conditioning TCM products, and supplements). The incidence of vertigo was higher in participants who were not menstruating (75.8%, *p*<.001), had endometrial polyps (8.6%, *p*=.006), and/or were under hormonal therapy (25.0%, *p*=.001; Table 2).

**Table 2:** Comparison of Health-Related Characteristics in Patients With and Without Vertigo

Characteristic	Total (*n*=1,423)	Vertigo (*n* =128, 9%)	Non-vertigo (*n*=1,295, 91%)	*p*
	*n* (%)	*n* (%)	*n* (%)	
Regular menstrual cycles				.483
No	225 (15.8)	23 (18.0)	202 (15.6)	
Yes	1,198 (84.2)	105 (82.0)	1,093 (84.4)	
Still menstruating				<.001
No	842 (59.2)	97 (75.8)	745 (57.5)	
Yes	581 (40.8)	31 (24.2)	550 (42.5)	
Period pain				.310
No	1,352 (95.0)	124 (96.9)	1,228 (94.8)	
Yes	71 (5.0)	4 (3.1)	67 (5.2)	
Uterine fibroids				.106
No	1,042 (73.2)	86 (67.2)	956 (73.8)	
Yes	381 (26.8)	42 (32.8)	339 (26.2)	
Ovarian cysts				.793
No	1,320 (92.8)	118 (92.2)	1,202 (92.8)	
Yes	103 (7.2)	10 (7.8)	93 (7.2)	
Endometriosis				.907
No	1,308 (91.9)	118 (92.2)	1,190 (91.9)	
Yes	115 (8.1)	10 (7.8)	105 (8.1)	
Endometrial polyps				.006
No	1,366 (96.0)	117 (91.4)	1,249 (96.4)	
Yes	57 (4.0)	11 (8.6)	46 (3.6)	
Previous use of hormone medications for ≥6 months ^a^				.001
No	1,207 (84.9)	96 (75.0)	1,111 (85.9)	
Yes	214 (15.1)	32 (25.0)	182 (14.1)	
Previous use of women’s health conditioning TCM products for ≥3 years ^b^				.790
No	1,212 (85.2)	108 (84.4)	1,104 (85.3)	
Yes	211 (14.8)	20 (15.6)	191 (14.7)	
Previous use of women’s health supplements for ≥3 months ^c^				.250
No	1,315 (92.4)	115 (89.8)	1,200 (92.7)	
Yes	108 (7.6)	13 (10.2)	95 (7.3)	

*Note.* TCM = traditional Chinese medicine.

^a^ Two records were missing. Including multivitamin/multimineral supplements.

^b^ Including traditional Chinese medicines such as harmonizing preventing miscarriage, health maintenance, nourishing blood for regulating menstruation, and menopause.

^c^ The primary women’s health supplements included evening primrose oil, placenta, and estrogen extracts.

**Figure 2:**
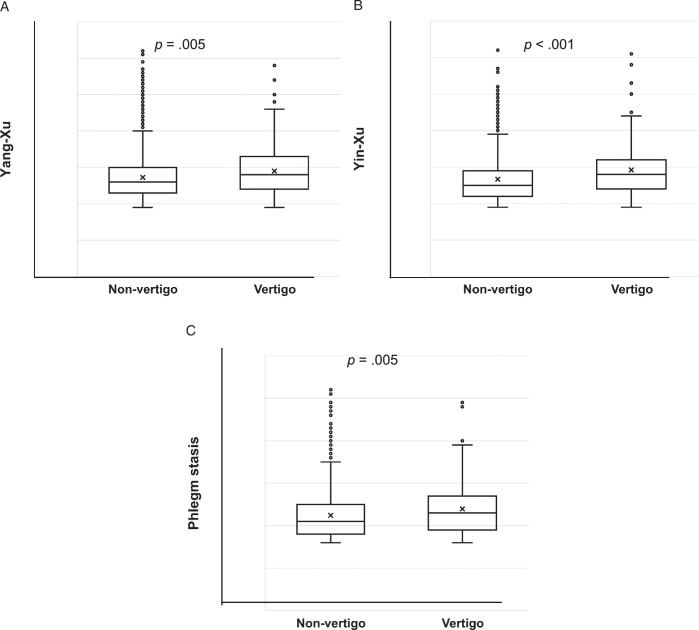
Relationships Between Various Constitutions in Patients With and Without Vertigo. *Note*. (A)–(C) show higher mean constitution scores across all constitution types in vertigo subjects. In the box plots, the cross is an average, the bold black line indicates the median per group, the box represents 50% of the values, the horizontal lines show minimum and maximum values of the calculated nonoutlier values, and the open circles indicate outlier values

Participant BCQ scores (mean±*SD*) are shown in Figure 2, with mean Yang-Xu, Yin-Xu, and Phlegm stasis scores of 27.41±6.71, 26.90±6.46, and 22.57±5.91, respectively. In terms of the relationship between BCQ scores for the three body constitutions and vertigo, it was found that, compared to those without vertigo, those with vertigo had a significantly higher means scores for all three, with Yang-Xu higher by 1.75 points (27.25±6.63 vs. 29.00±73.4, *p*=.005 respectively); Yin-Xu higher by 2.55 points (26.67±6.26 vs. 29.22±7.82, *p*<.001 respectively); and Phlegm stasis higher by 1.52 points (22.44±5.84 vs. 23.96±6.37, *p*=.005 respectively).

As shown in Table 3, forward stepwise analysis was used to explore the relationship between the three BCQ scores and vertigo, with the three BCQ scores and variables such as both sociodemographic and women’s health-related characteristics adjusted. Six factors were identified as significantly affecting vertigo, including the three scores of unbalanced constitutions (Yin-Xu, Yang-Xu, and Phlegm stasis), age, migraine, and endometrial polyps. In terms of body constitution, higher Yin-Xu, Yang-Xu, and Phlegm stasis scores were found to increase the odds of having vertigo by 1.033–1.049 per one-point increase in score (Yang-Xu: adjusted Odds Ratio [a*OR*]=1.033; 95% confidence interval [CI]=[1.007, 1.060]; *p*=.013, Yin-Xu: a*OR*=1.049; 95% CI=[1.022, 1.076]; *p*<.001, and Phlegm stasis: a*OR*=1.041; 95% CI=[1.010, 1.072]; *p*=.009). In terms of age, the odds of having vertigo increased by 1.057–1.060 for each additional year (*p*<.001); those with migraine had 1.564–1.676 higher odds of having vertigo (*p*=.015–.021); and those with endometrial polyps had 2.280–2.351 higher odds of having vertigo (*p*=.017–.022).

**Table 3:** Stepwise Logistic Forward Regression Analysis of Vertigo in Patients According to Body Constitution

Characteristic	Vertigo					
	Yang-Xu		Yin-Xu		Phlegm stasis	
	a*OR* [95% CI]	*p*	a*OR* [95% CI]	*p*	a*OR* [95% CI]	*p*
BCQ score ^a^	1.033 [1.007, 1.060]	.013	1.049 [1.022, 1.076]	<.001	1.041 [1.010, 1.072]	.009
Age (year)	1.058 [1.035, 1.081]	<.001	1.057 [1.034, 1.081]	<.001	1.060 [1.037, 1.083]	<.001
Migraine						
Yes versus no	1.676 [1.105, 2.543]	.015	1.564 [1.030, 2.375]	.036	1.640 [1.077, 2.498]	.021
Endometrial polyps						
Yes versus no	2.351 [1.166, 4.739]	.017	2.301 [1.131, 4.684]	.022	2.280 [1.128, 4.606]	.022

*Note*. a*OR* = adjusted odds ratio; CI = confidence interval; BCQ = Body Constitution Questionnaire. Adjusted for three types of BCQ scores (Yang-Xu, Yin-Xu, and Phlegm stasis) and sociodemographic characteristics (age, marital status, living status, education, work status, and BMI), metabolic syndrome (hypertension, hyperlipidemia, and diabetes), low back pain, migraine, and characteristics related to women's health (regular menstrual cycle, menstruation status, uterine myomas, ovarian cyst, endometriosis, endometrial polyps, previous use of hormone medications, previous use of TCM products for women’s health conditioning, and previous use of women's health supplements).

^a^ Score of BCQ is as follows: Yang-Xu, the total score of 19–95; Yin-Xu, 19–95; and Phlegm stasis, 16–80.

## Discussion

This study is one of only a few designed to explore the association between TCM body constitution and vertigo in Taiwanese women. In this retrospective study, the proportion of women with vertigo in the sample was 9.0%, and the mean age of onset was 56.23 years. The significant indicators of vertigo identified in the forward stepwise analysis showed that higher scores in Yin-Xu, Yang-Xu, and Phlegm stasis, together with migraine and endometrial polyps, were significant predictors of vertigo. The results of this study are expected to help TCM professionals and researchers strengthen their understanding of the acquired factors of TCM and provide a reference for future research.

Issues related to body constitution are often blamed for physical diseases (Li et al., 2019). By enhancing comprehension of and improving body constitution, the risk of numerous chronic conditions may be decreased, and TCM “preventive medicine” may be effectively implemented to boost patient health (Li et al., 2019). Liao et al. (2021) demonstrated a significant association between body constitution and chronic diseases such as hypertension, supporting unbalanced constitution as a risk factor for chronic conditions. In this study, imbalances in Yang deficiency, Yin deficiency, and Phlegm stasis were found to notably impact vertigo occurrence, underscoring the potential of body constitution influencing various health conditions. Consequently, interventions addressing unbalanced constitutions may facilitate chronic disease prevention.

According to TCM theory, Yang deficiency manifests as a lack of internal warmth, leading to the accumulation of bodily fluids and dampness, cold extremities, and poor blood circulation, which may result in vertigo (Maciocia, 1989; Yang, 2010). Yin deficiency, on the other hand, causes deficiency-heat syndrome, leading to symptoms such as fever, night sweats, and dryness, which disturb the mind and cause vertigo. Furthermore, it results in insufficient nourishment of the brain and sensory organs, leading to vertigo (Maciocia, 1989; Yang, 2010). A study conducted in Hong Kong reported Yin-Xu may be associated with chronic stress and long working hours, which may cause fatigue, weakness, vertigo, and susceptibility to contracting colds (L. Guo et al., 2022). Phlegm stasis is mainly attributable to the slowing or insufficient interaction of yin and yang energies, resulting in unfavorable qi and blood, and vertigo and discomfort, which are more common in postmenopausal women (P.-H. Chen et al., 2020; Maciocia, 1989; Yang, 2010). Overall, vertigo results from the complex interplay of deficiencies and excesses. Yang deficiency leads to fluid retention and Phlegm formation, Yin deficiency leads to increased internal heat that disrupts fluid balance, and Phlegm stasis further exacerbates Yang and Yin deficiencies.

Western medication treatments for vertigo are associated with limited efficacy and side effects (Oh et al., 2022). In contrast, TCM treats vertigo based on patient-specific symptoms and clinical manifestations (Oh et al., 2022), prescribing treatments that primarily involve warming Yang to activate collaterals, nourishing Yin to moisten dryness, and dissipating Phlegm to restore balance and alleviate vertigo (Z. Guo et al., 2017; Ma et al., 2021). In addition, vertigo may be effectively treated through the application of a layered needling technique at Zhongwan and “gate points” in the neck region, along with the tendon-bone needling technique utilizing the modified “dark tortoise seeking hole” at local tendon blockage points. This approach targets the regulation of qi in the middle jiao, opening of the gate, nourishing of the marrow, relaxation of the tendon, and harmonization of the mind (S.L. Wang et al., 2022). Reactions of the ear can regulate the qi and blood of the internal organs, harmonize yin and yang, dredge the meridians, and invigorate the kidneys and spleen, consequently achieving the desired effects of vertigo suppression (C. Liu et al., 2022).

Consistent with the findings of previous studies, the odds of developing vertigo were found to increase by 1.057–1.060 for every 1-year increase in age (Agrawal et al., 2018; Kovacs et al., 2019; Wassermann et al., 2022). Wassermann et al. (2022) found that vertigo risk changes with age; vertigo episode duration is higher in the elderly; and the frequency of daily vertigo symptoms is ~15% higher in the elderly than in younger patients. H. W. Lin and Bhattacharyya (2012) investigated the prevalence of vertigo and balance disorders among older adults in the United States, finding that 30% of adults ≥65 years old had experienced vertigo during the prior 12-month period and that older women were more susceptible than older men (21% vs. 18%). Thus, the incidence of vertigo increases and becomes more frequent with age. The findings of this study also indicate that women with migraine symptoms are more likely to experience vertigo, which is similar to the findings of Oğuz-Akarsu et al. (2020).

Chronic migraine attacks in women are associated with many symptoms, including nausea, vomiting, osmotic phobia, vertigo, and allodynia, and may be accompanied by dysautonomia (e.g., nausea, vomiting, and vertigo) and sensory symptoms (e.g., photophobia, phonophobia, and skin allodynia; Cocores & Monteith, 2022). Estrogen directly affects the trigeminal nervous system. The frequency of experiencing vertigo during menstruation is higher in women who suffer from migraines during menstruation and decreases after menopause, which is attributed to the complex relationship between estrogen and migraine, influenced by fluctuations in gonadal hormone levels (Godley et al., 2024).

The potential links between endometrial polyps and vertigo have been rarely explored, and no clear evidential support is available suggesting a direct association between endometrial polyps and vertigo (Nijkang et al., 2019). In this study, 8.6% of the participants had both endometrial polyps and vertigo, and multivariate logistic regression analysis identified women with endometrial polyps at a 2.28–2.35-times higher risk of experiencing vertigo. In addition, more than 80% of the women in this study reported irregular menstrual cycles, which may reflect underlying hormonal dysregulation associated with endometrial polyps. Previous studies have reported that endometrial polyps can irritate the surrounding tissue, leading to spotting or vaginal bleeding and signs such as irregular menstrual bleeding, heavy bleeding, and postmenopausal vaginal bleeding symptoms that promote fatigue, weakness, shortness of breath, pale skin, and vertigo, and affect ~68% of pre-and postmenopausal women (Kovacs et al., 2019; Nijkang et al., 2019). Within the TCM framework, polyps are related to an unbalanced constitution, usually a combination of blood deficiency, blood stagnation, and liver/kidney deficiency (Zhu et al., 2018). Endometrial polyps may cause menorrhagia, blood deficiency, liverzang-fire attacking lungs, vertigo, insomnia, and other symptoms (Zhu et al., 2018). Hysteroscopic surgery, while an effective method of removing endometrial polyps, is not always successful, which, under TCM theory, is because the underlying cause of polyps has not been addressed, resulting in an internal environment of dampness, heat, and blood stasis in the uterus (B. Q. Liu & Cheng, 2013). The results of this study support women with vertigo symptoms to undergo further gynecological examinations as a reference for improved diagnosis.

### Limitations

In this study, types of vertigo were not distinguished, and vertigo diagnosis details were not available, which may affect the objectivity of the results, necessitating further exploration of the relationship between vertigo types and TCM constitution. Despite these limitations, the secondary data used in this study were obtained from the TWB, the largest population-based database in Taiwan, from which we extracted information on participants’ diseases and medication use. The results provide medical institutions with a clearer understanding of the underlying factors that affect vertigo in women.

### Conclusions

In this study, vertigo risk in women was found to be influenced by age, migraine, and endometrial polyps. Furthermore, the results of the BCQ assessment showed an unbalanced constitution to be associated with a higher risk of vertigo in women. Body constitution refers to an individual’s overall physical and mental state, while pattern recognition depends on the physical condition and disease being treated. However, the role of the body constitution in the occurrence and treatment of vertigo in women requires further research.
